# Contribution of Hepatitis C Infection to a Large Cohort of Cryoglobulin-Positive Patients: Detection and Characteristics

**DOI:** 10.3389/fimmu.2020.01183

**Published:** 2020-06-30

**Authors:** Marie N. Kolopp-Sarda, Pierre Miossec

**Affiliations:** ^1^Immunogenomics and Inflammation Research Unit EA 4130, University of Lyon, Lyon, France; ^2^Immunology Laboratory, Hospices Civils de Lyon, Lyon, France; ^3^Department of Immunology and Rheumatology, Clinical Immunology Unit, Hospices Civils de Lyon, Lyon, France

**Keywords:** cryoglobulin, hepatitis C, rheumatoid factor, complement, immunoglobulin

## Abstract

Cryoglobulins (CGs) are cold precipitating immunoglobulins, and hepatitis C virus (HCV) infection is its most common cause. The purpose of the study was to determine the contribution of HCV in a large cohort of CG. Biological characteristics and specificity of CGs in HCV patients were compared to non-HCV subjects. Cryoglobulin analysis included isotype, clonality, concentration, and rheumatoid factor (RF) in cryoprecipitate and serum complement and RF. This study is an extension of the study carried out on a cohort of 13,439 patients tested for CGs from all medical units, in which 1,675/13,439 (12.5%) patients had a CG, and 680/1,675 (40.6%) had HCV serology or viral load determination (HCV RNA). Among these 680 CG patients tested for HCV, 325 of 680 (47.8%) HCV patients (272 HCV RNA^+^ and 45 HCV RNA^−^ patients) were compared to 355/680 (52.2%) non-HCV subjects. After a positive detection of CG, HCV status was determined only for 37.7% (256/680) of patients, allowing the diagnosis of a previously unknown HCV infection for 39.8% (102/256). Concentration of HCV RNA^+^ CGs (median = 80.5 mg/L) was significantly higher than that of HCV RNA^−^ CG (median = 50.5 mg/L, *p* = 0.001) and HCV^−^ CG (median = 32 mg/L, *p* < 0.0001). There was no difference of median CG concentration between HCV RNA^−^ patients and non-HCV subjects. Rheumatoid factor titer was significantly higher in type II CG compared to type III CG in HCV RNA^+^ patients (254 ± 720 vs. 15 ± 21 IU/mL, *p* < 0.0001) and non-HCV subjects (333 ± 968 vs. 16.8 ± 26 IU/mL, *p* = 0.0004). Complement functional activity CH50 was lower in HCV RNA^+^ patients (36 ± 24 U/mL) and in HCV RNA^−^ patients (32 ± 21 U/mL) than in non-HCV subjects (50 ± 25 U/mL, *p* = 0.001 and *p* = 0.004). In conclusion, HCV infection and treatment influence CG characteristics. It is essential, and far from always tested, to determine the HCV status of patients with mixed CG, and conversely to search for CG in patients with HCV infection.

## Introduction

Cryoglobulins (CGs) are immunoglobulins (Ig) precipitating at cold temperature and dissolving at 37°C. They are classified in 3 types (1): type I CG composed of a monoclonal Ig, type II of a monoclonal Ig with rheumatoid factor (RF) activity bound to polyclonal IgG, and type III, of polyclonal Ig. Types II and III CG are called mixed CG. Type I CGs are secondary to lymphoproliferative diseases. Mixed CGs are secondary to infections and autoimmune diseases (2).

Before 1990, most mixed CGs were defined as essential CG. As there was a high frequency of liver involvement, association with hepatitis B virus (HBV) infection was suspected, but with no direct proof of HBV involvement, especially no evidence of HBV virus in the cryoprecipitate (3). Hepatitis C virus (HCV) was discovered later (4) and was identified in type II CGs isolated from HCV serum, highlighting the direct involvement of HCV in CGs synthesis (5–7). Many essential type II CGs were then classified as secondary CGs to HCV infection (6–8).

Mixed CGs in HCV patients can contribute to extrahepatic manifestations, including purpura, vasculitis, renal involvement, and arthralgia (9–13). Pathophysiology of CGs in HCV context can result from the formation of immune complexes composed of an IgM RF associated with specific anti-HCV IgG, which precipitate in small and medium size vessels (14–16).

The biological characteristics of CGs in this population compared with HCV-negative population have not been fully described. In a previous study of a very large cohort of patients, mixed CGs were mainly isolated in HCV patients (49.5%), compared with other chronic viral infection (HBV and HIV) and autoimmune diseases (14). The present study is an extension of the previous study, with focus on HCV infection. The aims of this study were to compare the biological characteristics of CGs with and without HCV infection and to evaluate how often HCV detection was done in CG-positive patients and conversely for the detection of CGs in HCV patients.

## Materials and Methods

### Study Population and CG Purification and Characterization

This study was a retrospective study conducted at the University Hospitals of Lyon (France). From January 2010 to December 2016, 13,439 patients had at least one serum sample for CG detection sent to the central immunology laboratory (mean age = 52.4 ± 17.9 years; 8,026 women and 5,413 men, female-to-male ratio = 1.48) (14). Patients with positive CG detection and hepatitis C serology and/or viral load determination were included in this series. The protocol was approved by the Ethics Committee of the Hospitals of Lyon for the protection of people participating in biomedical research under the number AC-2016-2729.

Hepatitis C viral humoral response was measured by two techniques [Monolisa anti-HCV Plus V2 (Bio-Rad, Marnes-la-Coquette, France), ADVIA Centaur® HCV assay, and Enzygnost Anti-HCV 4.0 (Siemens Healthcare Diagnostics, Marburg, Germany)] and validated on two samples. Active viral infection was measured by HCV viral load and assayed by quantitative HCV RNA analysis (RealTime HCV; Abbott Laboratories, Des Plaines, IL, USA). The course of disease and the effectiveness of antiviral treatments are monitored by measuring the viral load.

All CGs were detected and analyzed with the previously described techniques (2, 14). Blood samples were collected in 3- to 5-mL tubes with red top and clot activator (BD Vacutainer, Franklin Lakes, NJ, USA). After sampling, tubes were maintained in an incubator at 37°C for 2 h to clot and to avoid CG precipitation. Once clotted, the samples were centrifuged for 15 min at 2,200 g. Sera were decanted in conical bottom tubes, and these tubes were kept at +4°C for 7 days. A 500 μL aliquot of serum was identically treated and used for serum RF. Visual observation at day 7 allows the detection of a cryoprecipitate. The cryoprecipitate was isolated by cold centrifugation (15 min, 2,200 g, 4°C) and purified using three washes with cold (4°C) phosphate-buffered saline (PBS) (pH 7.4). After each wash, the samples were centrifuged at 4,500 g for 15 min at 4°C. Following the last wash, ≥500 μL PBS (function of the precipitate volume) was added to the pellet, with Fluidil 2% (Sebia, Lisses, France) to fluidify the suspension and facilitate precipitate dissolution. This sample was vortexed and placed at 37°C for ≥2 h to dissolve the precipitate for further analysis.

The CGs were identified by immunofixation–electrophoresis of the dissolved cryoprecipitate with antisera anti-γ, anti-α, anti-μ, anti-κ, and anti-λ (SAS-3; Helena Biosciences, Gateshead, UK) and classified according to their Ig monoclonal and/or polyclonal profile (1). The IFE indicated the degree of purity of the cryoprecipitate by the absence of other serum proteins. The identified Ig's were quantified by immunonephelometry with reagents for low concentrations (BN ProSpec; Siemens). The concentration of Ig in the cryoprecipitate was adjusted to the initial volume of serum from which the CGs were isolated, and the results were expressed as milligrams per liter of serum. For mean and median comparison, CG concentration was expressed as total Ig concentration in the cryoprecipitate.

### Additional Analysis of the Cryoprecipitate and Serum

The RF activity was measured in the cryoprecipitate and in a serum aliquot by immunonephelometry (BN ProSpec, Siemens).

Complement functional activity (CH50) was measured using Optilite (The Binding Site, St Egrève, France) and C3 and C4 fraction concentration using BN ProSpec, in the serum sample obtained at the same time as CG detection. Reference ranges in our laboratory are 0.82–1.70 g/L for C3, 0.14–0.32 g/L for C4, 41–95 U/mL for CH50.

### Statistical Analysis

For continuous variables, the results were expressed as mean ± SD or as the median (range). Distribution normality was tested using the D'Agostino Pearson omnibus normality test. The Kruskal–Wallis test was used for the variance analysis and the Mann-Whitney *U*-test for the comparison of quantitative variables. The Student *t*-test and Wilcoxon test were used for the comparison of quantitative parameters. The Fisher exact test was used to analyze qualitative differences. *P* < 0.05 was considered statistically significant. Calculations were performed with GraphPad Prism software version 5.01 (GraphPad Prism, La Jolla, CA, USA).

## Results

### Study Population

**Figure 1:**
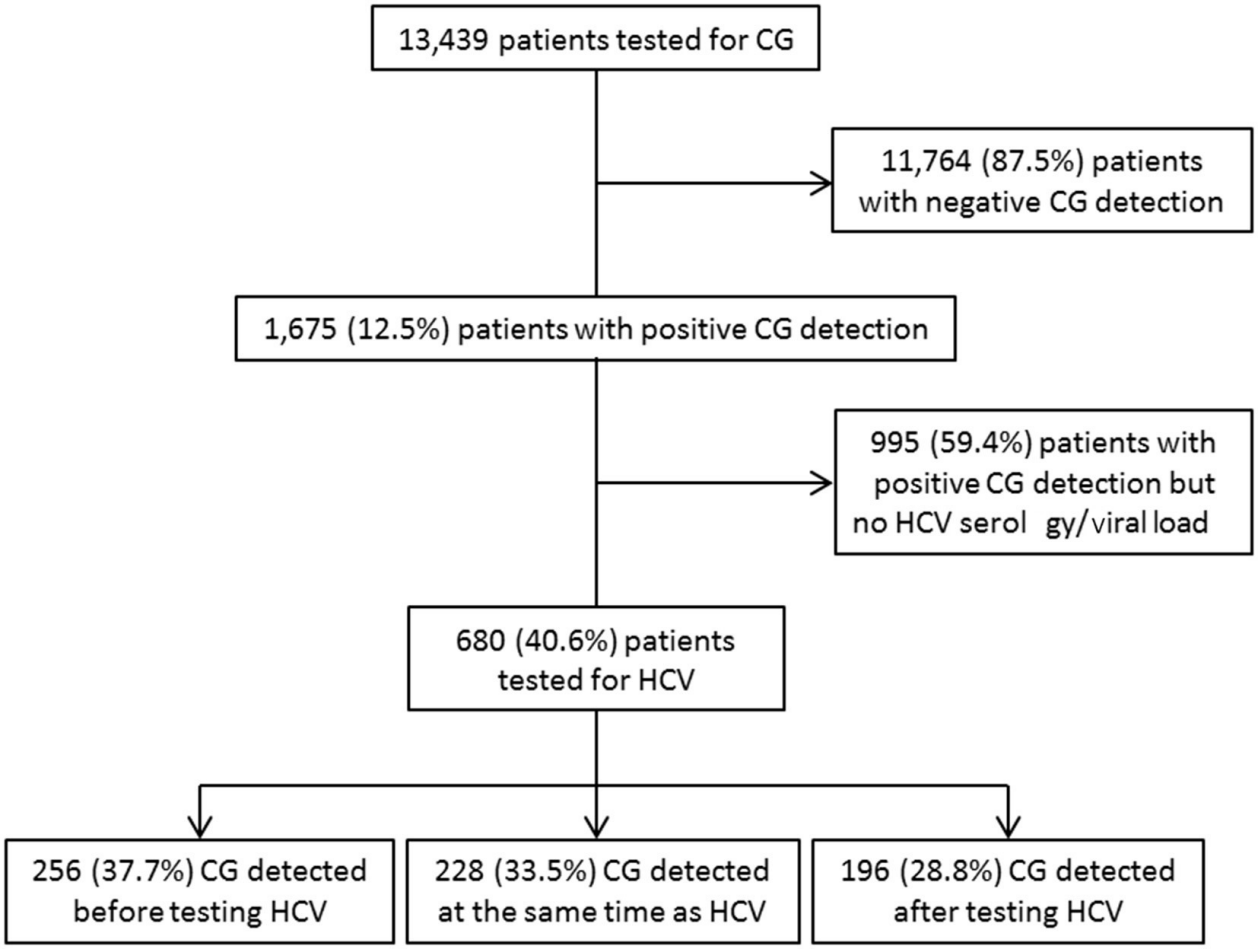
Study flowchart of patient inclusion within the study period (2010–2016) and relation with HCV status determination.

Of a cohort of samples from 13,439 patients analyzed for CG detection, 1,675 patients (12.5%) had a positive detection of CGs (14). Although HCV is a well-known cause of CG, 995/1,675 (59.4%) CG^+^ patients had no determination of their HCV status. For 680/1,675 (40.6%) patients with CG, HCV status was known: there were 355 HCV-negative subjects (negative serology, non-HCV subjects) and 325 HCV-positive patients (HCV patients) included in this study (Figure 1). Among the 325 HCV patients, 317/325 (97.5%) had a positive serology associated with an HCV RNA measurement, 8/325 (2.5%) had no HCV RNA determination. There was a positive serology associated with a detectable viral load (>15 UI/mL, HCV RNA^+^ patients) for 272/317 (85.8%) patients and a positive serology associated with a non-detectable RNA (HCV RNA^−^ patients) for 45/317 (14.2%) patients. For the 45 HCV RNA^−^ patients, 43/45 (95.6%) were treated patients at the moment of CG sample, and 2/45 patients were not treated because of weak antibody titer and negative viral load (Table 1).

**Table 1:** Demographic, HCV status, and CG characteristics of included patients.

**Patient characteristics**	**Non-HCV subjects**	**HCV patients**	**HCV RNA^**+**^ patients**	**HCV RNA^**−**^ patients**	***P***
*n*	355	325	272	45	
Age, mean ± SD, years	53.6 ± 12.4	56.2 ± 12.4	56.7 ± 12.8	54.7 ± 10.3	0.03^§^
Sex ratio	207 F/148 M	150 F/175 M	135 F/137 M	12 F/33 M	
	(F/M = 1.39)	(F/M = 0.86)	(F/M = 0.99)	(F/M = 0.36)	0.002^§^
HCV viral load measurement (*n*)		317/325 (97.5%)	272	45	
CG type					
Type I CG	28/355 (7.9%)	4/325 (1.2%)	4/272 (1.5%)	0/45 (0%)	<0.0001^§^
Type II CG	155/355 (43.7%)	153/325 (47.1%)	127/272 (46.7%)	21/45 (46.7%)	0.95^§^/0.99^§§^
Type III CG	172/355 (48.4%)	168/325 (51.7%)	141/272 (51.8%)	24/45 (53.3%)	0.95^§^/0.99^§§^
Concentration, median (range), mg/L	32 (7–13,429)	79 (10.9–9,440)	80.5 (10–9,440)	50.5 (13–344)	<0.0001^§^/0.001^§§^
Rheumatoid factor (IgM anti-IgG)					
Type II CG RF^+^	43/155 (27.7%)	35/153 (22.9%)	29/127 (22.8%)	3/21 (14.3%)	0.36^§^/0.34^§§^
Type III CG RF^+^	17/172 (9.9%)	28/168 (16.7%)	24/141 (17%)	4/24 (16.7%)	0.07^§^/0.34^§§^
RF titer, median (range), IU/mL	18 (3–4,903)	12 (2–8,267)	12 (2–3,548)	ND	<0.0001^§^
Complement exploration					
C3, g/L	1.00 ± 0.36	1.08 ± 0.24	1.09 ± 0.24	1.05 ± 0.26	0.13^§^/0.11^§§^
C4, g/L	0.20 ± 0.13	0.19 ± 0.09	0.19 ± 0.10	0.17 ± 0.07	0.43^§^/0.38^§§^
CH50, U/mL	50 ± 25	35 ± 23	36 ± 24	32 ± 21	0.004^§^/0.44^§§^

§ *Comparison between non-HCV patients and HCV patients*.

§§ *Comparison between HCV RNA^+^ patients and HCV RNA^−^ patients*.

*RF, rheumatoid factor; ND, not done (too small group)*.

Among these 680 CG^+^ patients, 256/680 (37.7%) had the determination of HCV status after the CG detection, and this allowed the diagnosis of a previously unknown HCV infection for 102/256 (39.8%) patients. For 196/680 (28.8%) patients, the HCV status led to a CG detection. For 228/680 (33.5%) patients, HCV infection and CGs were detected at the same time (Figure 1).

Demographic and immunological characteristics of CGs in patients with known HCV status are reported in Table 1. In the group of HCV patients, there were more men than women (150 women and 175 men, female-to-male ratio = 0.86), compared to the non-HCV subjects (207 women and 148 men, female-to-male ratio = 1.39, *p* = 0.002). Hepatitis C virus patients were older than non-HCV subjects (mean age = 56.2 ± 12.4 vs. 53.6 ± 12.4 years, *p* = 0.03).

**Figure 2:**
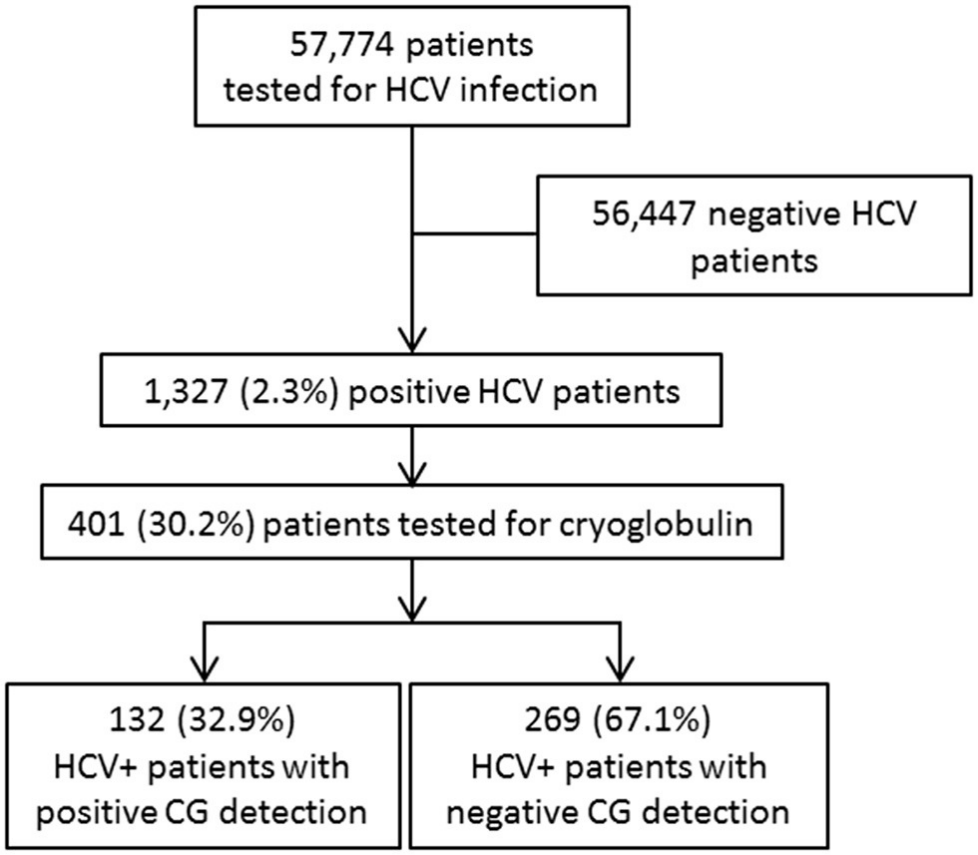
Hepatitis C virus infection determination and CG detection.

Because of the important association of HCV and CG, it was interesting to evaluate the number of patients with an HCV infection for whom CGs were detected. In the same university hospital, 57,774 HCV serology detections were performed from 2013 to 2016; 1,327 patients had an HCV-positive serology (2.3%); CG was searched for 401 of them (30.2%); and positive for 132/401 (32.9%) (Figure 2).

### Cryoglobulin Type and Concentration

Among the 325 HCV patients, CG types were divided into 4/325 (1.2%) type I CGs (all IgM kappa), 153/325 (47.1%) type II CGs (80 IgM kappa, 35 IgM lambda, 29 IgG kappa, and 9 IgG lambda, all associated with polyclonal IgG/IgM and/or IgA), and 168/325 (51.7%) type III CGs (154 IgG/IgM, 8 IgG, 5 IgM, 1 IgG/IgA). Among the 355 non-HCV subjects, there were 28/355 (7.9%) type I CGs (9 IgM kappa, 7 IgM lambda, 6 IgG kappa, and 6 IgG lambda), 155/355 (43.7%) type II CGs (100 IgM kappa, 25 IgM lambda, 16 IgG kappa, 11 IgG lambda, and 3 IgA kappa, all associated with polyclonal Ig), and 172/355 (48.4%) type III CGs (127 IgG/IgM, 15 IgG, 11 IgM, and 19 IgG/IgA/IgM). There was no difference in the distribution of types II and III CGs between the two groups (*p* = 0.95). As expected, type I CGs were more frequent in the non-HCV compared to HCV patients (*p* < 0.0001).

Four of 272 (1.5%) HCV RNA^+^ patients had type I CG, 127/272 (46.7%) had type II CGs (of which 29 RF^+^ CG), and 141/272 (51.8%) had type III CGs (of which 24 RF^+^ CG). The four patients with type I CG in this group have been monitored for their hepatitis for more than 5 years post-treatment, and none of them has yet developed a lymphoproliferative disease.

In the group of HCV RNA^−^ patients, 21/45 (46.7%) had type II CGs (of which three RF^+^ CGs), and 24/45 (53.3%) had type III CGs (of which four RF^+^ CGs). There was no difference in the distribution of types II and III CGs between HCV RNA^+^ and HCV RNA^−^ patients (*p* = 0.99).

The median CG concentration in HCV RNA^+^ patients (80.5 mg/L; range = 10–9,440) was significantly higher than in HCV RNA^−^ patients (50.5 mg/L; range = 13–344, *p* = 0.001) and in non-HCV subjects (32 mg/L; range = 7–13,429; *p* < 0.0001). There was no difference of median CG concentration between HCV RNA^−^ patients and non-HCV subjects.

**Figure 3:**
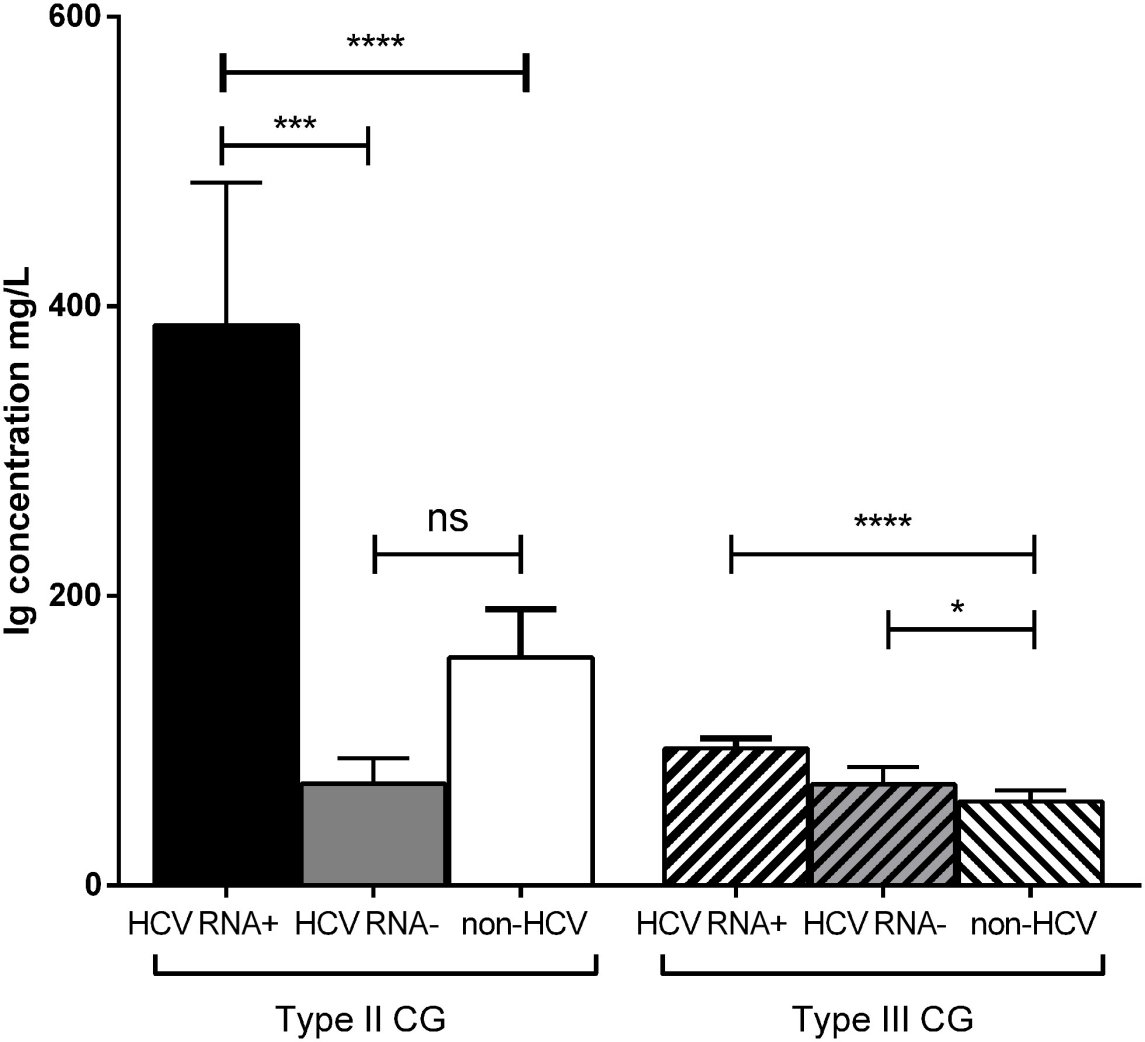
Concentration of types II and III CG in HCV patients and non-HCV subjects. Three groups were compared: 272 HCV RNA^+^ patients, 45 HCV RNA^−^ patients, 355 non-HCV subjects (mean ± SEM, ****p < 0.0001, ***p = 0.001, *p = 0.03). ns, not significant.

Type II CG concentration was significantly higher in HCV RNA^+^ patients (387 ± 1,110 mg/L) than in HCV RNA^−^ patients (70.4 ± 78.3 mg/L, *p* = 0.001) and in non-HCV subjects (157 ± 411 mg/L, *p* < 0.0001). There was no difference of type II CG concentration between HCV RNA^−^ patients and non-HCV subjects (Figure 3).

Type III CG concentration was significantly higher in HCV RNA^+^ patients (94.7 ± 83.8 mg/L, *p* < 0.0001) than in non-HCV subjects (58 ± 105 mg/L) but not than that in HCV RNA^−^ patients (69.9 ± 58.8 mg/L, *p* = 0.62). Type III CG concentration was significantly higher in HCV RNA^−^ patients than in non-HCV subjects (*p* = 0.03; Figure 3).

### Rheumatoid Factor Activity

There was no difference for the presence of RF in type II CGs from non-HCV subjects compared with HCV patients [27.7% (43/155) vs. 22.9% (35/153), *p* = 0.36], and in type III CGs between these two groups [9.9% (17/172) vs. 16.7% (28/168), *p* = 0.07; Table 1]. Among the HCV patients, there was no difference in the proportion of RF^+^ type II CGs between HCV RNA^+^ [29/127 (22.8%)] and HCV RNA^−^ [3/21 (14.8%), *p* = 0.34] patients, and in the proportion of RF^+^ type III CGs between HCV RNA^+^ [24/141 (17%)] and HCV RNA^−^ [4/24 (16.7%), *p* = 0.34] patients. But in non-HCV subjects, RF^+^ type II CGs were more frequent than RF^+^ type III CGs (27.7% vs. 9.9%, *p* < 0.0001; Table 1).

**Figure 4:**
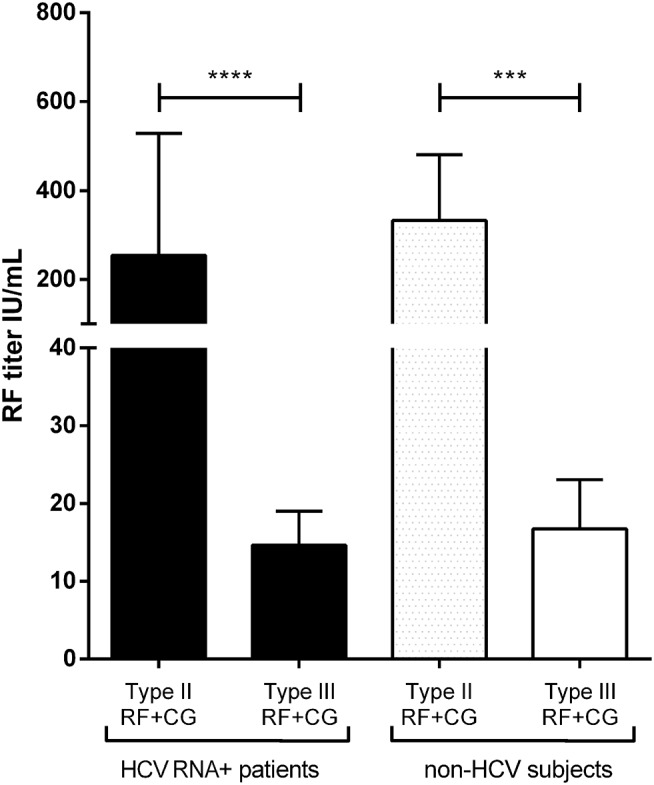
Quantification of RF in HCV RNA^+^ CG and non-HCV CG. Type II CG (29 HCV RNA^+^ patients and 43 non-HCV subjects) and type III CG (24 HCV RNA^+^ patients and 17 non-HCV subjects) (mean ± SEM, ****p < 0.0001; ***p = 0.0004).

Rheumatoid factor quantification showed a higher titer in HCV RNA^+^ RF^+^ type II CG (254 ± 720 IU/mL) compared to HCV RNA^+^ RF^+^ type III CGs (15 ± 21 IU/mL, *p* < 0.0001). In non-HCV subjects, type II CGs RF titer (333 ± 968 IU/mL) was higher than type III CGs RF titer (16.8 ± 26 IU/mL, *p* = 0.0004). There was no difference in type II CGs RF titer between HCV RNA^+^ patients and non-HCV subjects (*p* = 0.59), as in type III CGs RF titer in HCV patients and in non-HCV subjects (*p* = 0.52; Figure 4).

### Immunoglobulin Isotype Concentration in Mixed CGs With RF Activity

Most mixed CGs are composed of monoclonal (type II) or polyclonal (type III) IgM, often bound to polyclonal IgG through RF activity. Concentration of each isotype in the cryoprecipitate in RF^+^ CGs could give information on IgG–IgM association in types II and III CG. In HCV patients, there was no difference between IgG and IgM concentration in type II RF^+^ CGs (IgG = 345 ± 504 mg/L and IgM = 634 ± 1,120 mg/L, *p* = 0.17) and in type III RF^+^ CGs (IgG = 68.6 ± 35.5 mg/L and IgM = 80.9 ± 80.9 mg/L, *p* = 0.46). In non-HCV subjects, there was also no difference between IgG and IgM concentration in type II RF^+^ CGs (313 ± 761 mg/L and 473 ± 988 mg/L, *p* = 0.40) and in type III RF^+^ CG (35.3 ± 30.2 and 57.7 ± 84.5 mg/L, *p* = 0.32).

### Complement Exploration

Complement (C3 and C4 fractions, and CH50 functional activity) was explored in the serum of 42/272 (15.4%) HCV RNA^+^ patients, 15/45 (33.3%) HCV RNA^−^ patients, and 231/355 (65%) non-HCV subjects, together with CG detection. Decrease of C4 (C4 ≤ 0.12 g/L) associated or not with decrease of C3 (C3 ≤ 0.80 g/L) was found in 24.5% (10/42) of HCV RNA^+^ patients, 26.7% (4/15) of HCV RNA^−^ patients, and 30.7% (71/231) of non-HCV subjects (χ^2^-test, *p* = 0.88).

C3 concentration was not significantly different between HCV RNA^+^ patients (1.09 ± 0.24 g/L), HCV RNA^−^ patients (1.05 ± 0.26 g/L), and non-HCV subjects (1.0 ± 0.36 g/L). There was no more significant difference for C4 concentration between HCV RNA^+^ patients (0.19 ± 0.1 g/L), HCV RNA^−^ patients (0.17 ± 0.07 g/L), and non-HCV subjects (0.2 ± 0.13 g/L). But functional activity measured by CH50 was significantly lower in HCV RNA^+^ patients (36 ± 24 U/mL) and HCV RNA^−^ patients (32 ± 21 U/mL) than in non-HCV subjects (50 ± 25 U/mL, *p* = 0.001 and *p* = 0.004; Table 1), in relation with complement classical pathway activation and not only fixation of these fractions within the immune complexes.

## Discussion

This study is an extension of the study carried out on a cohort of 13,439 patients tested for CGs from all medical units, focusing on HCV infection and its contribution in CGs immunological characteristics. This study describes the biological characteristics of CGs in HCV compared to non-HCV patients. It also highlights too common the lack of detection of CGs in HCV patients and the lack of diagnosis for HCV infection in mixed CG-positive patients. Hepatitis C virus RNA^+^ CGs had a higher concentration than HCV RNA^−^ CGs and non-HCV CG. Cryoglobulin with RF activity was found in the same proportion in HCV patients and non-HCV subjects. Complement system was more activated in HCV RNA^+^ patients and HCV RNA^−^ patients, by CG immune complexes, as shown by a decrease of CH50.

Demographic characteristics of this HCV population showed a higher proportion of men than in non-HCV subjects, as found in the whole cohort (14). This is explained by the higher frequency of men infected by HCV (63% in 2013 and 56% in 2016, with median age 51 years in the French LaboHep study) (17, 18).

The strong link between HCV infection and CGs was demonstrated in 1990 (6, 9, 19–21). It is thus very important to determine HCV status in patients with CG and conversely look for the presence of a CGs in HCV patients. However, 38% of CG^+^ patients had a determination of HCV infection, and it was detected in 40% of these patients. Cryoglobulin detection in a context of vasculitis is a good way to detect chronic HCV infection (10, 11). Only 30% of HCV-infected patients had a CG detection. Detection of CGs in HCV patients is not systematically done with figures ranging from 40 to 66% (9, 10, 12, 14, 22). It is essential to detect CGs in HCV patients at the time of viral diagnosis. The presence of a CG, specifically of type II, is the first sign of a monoclonal component that may later develop into a B-cell lymphoma.

Presence of a detectable viral load is a marker of active infection, and in HCV patients with positive viral load, type II CGs were detected in higher concentration than type II CGs in patients without detectable RNA. These results are consistent with the pathophysiologic mechanisms of type II HCV^+^ CG, in presence of HCV virions. Immune complexes formed by cryoprecipitating monoclonal RF IgM recognizing anti-HCV IgG precipitate in small-sized vessels when temperature decrease (6, 16, 20, 23). Cryoglobulin concentration in HCV-treated patients was not different from that of non-HCV subjects, where CG was secondary to autoimmune diseases or other infections.

Decrease of complement functional activity in HCV patients is an additional argument for immune complexes formation and complement classical pathway activation. Type III CGs in HCV patients had a very low concentration and low RF titer, explaining why type III CGs in HCV patients are less pathogenic than type II CGs (6, 16). This pathophysiological hypothesis needs to be confirmed in a clinical study of this cohort.

The combination of IgG and IgM in the cryoprecipitate of RF-positive CGs could reflect the RF activity of pentameric IgM recognizing from 2 to 5 IgG when temperature decreases (24). This could not be demonstrated by Ig isotype measurement in the cryoprecipitate because there were no differences in IgG and IgM concentrations in RF^+^ CG. The comparable concentrations of IgM and IgG, instead of the higher IgG concentration than IgM, could be due to the presence of different forms of polymerization of IgM molecules (from monomers to pentamers). This is linked to modifications in Ig glycosylation and amino acid composition (25, 26). These modifications are frequently described in the case of cryoprecipitating IgM (27).

The presence of a CG increases the risk of extrahepatic and tumoral manifestations in HCV patients, and their detection is essential for treatment selection and follow-up (28–30). The use of sensitive techniques of concentration measurement and detection of RF activity in the cryoprecipitate are critical. For the Ig concentration in the cryoprecipitate, immunonephelometry was used rather than cryocrit measurement, which has poor sensitivity and specificity. This is critical for detection and monitoring of mixed CG, which could have a low concentration compared to type I CGs associated with lymphoproliferative diseases.

Moreover, RF activity was measured in the cryoprecipitate, which allowed specific detection of cryoprecipitating anti-IgG IgM, and not serum RF (14–16, 31).

## Conclusion

This study of the characteristics of HCV-associated CGs in a very large cohort demonstrated the importance of the detection of CGs in all HCV patients and the detection of HCV infection in all CG patients. The characterization of CGs in this context is useful for the diagnosis of extrahepatic clinical manifestations, the implementation of specific treatments, and their follow-up.

## Data Availability Statement

The datasets generated for this study are available on request to the corresponding author.

## Ethics Statement

This protocol was approved by the Ethics Committee of the Hospitals of Lyon for the protection of persons participating in biomedical research under number AC-2016-2729.

## Author Contributions

MK-S: study and first draft. PM: concept of study and revision. All authors contributed to the article and approved the submitted version.

## Conflict of Interest

The authors declare that the research was conducted in the absence of any commercial or financial relationships that could be construed as a potential conflict of interest.

## Abbreviations

CG: cryoglobulin(s)
C3, C4: complement fractions 3 and 4
CH50: functional activity of the complement system
HBV: hepatitis B virus
HCV: hepatitis C virus
HIV: human immunodeficiency virus
Ig: immunoglobulin
RF: rheumatoid factor.
